# Paroxysmal Œdema of the Lungs

**Published:** 1913-06

**Authors:** C. E. S. Flemming


### PAROXYSMAL ŒDEMA OF THE LUNGS.

BY

C. E. S. FLEMMING, M.R.C.S. Eng., L.R.C.P. Lond.

ACUTE œdema of the lungs, better called paroxysmal œdema, is a condition treated by the text-books in the most cavalier manner, though it is a very serious and probably a not very infrequent affection. In twenty-seven years' private practice

I have recognised four cases, and may possibly have overlooked others. I will give as concisely as possible an account of the signs and symptoms common to my cases.

The attack most often commences with some irritation in the throat, tickling and feeling of dryness with slight cough, and the patient has a vague fear of some serious happening. In later attacks this fear amounts almost to terror. He cannot, dare not, lie down ; breathing at once becomes rapid, the face looks anxious and is pale. There is generally, but not always, some cyanosis with the pallor. The skin is cold and moist ; there is a sense of suffocation ; breathing is oppressed and urgent ; cough grows more distressing, and there is some expectoration of frothy coagulable fluid, which is frequently blood-stained ; the chest is rapidly filled with noisy râles ; the pulse is small and quick ; breathing becomes more distressing ; expectoration at times becomes more profuse, but this is not by any means always the case. This condition continues for perhaps half an hour or an hour and a half. Gradually the breathing becomes deeper, easier and quieter, the pulse is fuller and less rapid, the skin resumes its normal colour and warmth ; the patient, though exhausted, is thankful for the relief, but too nervous of a return of the symptoms to sleep soundly. Air now enters the chest freely, but râles persist over the greater part of one or both lungs. In one or at most two days all physical signs of the œdema have disappeared, and the attack is a thing of the past, to return, however, it may be in a week or two, it may be not for months. In one case the second attack was fatal ; in another attacks recurred at irregular intervals for more than two years, the patient dying eventually of heart-failure following shingles.

The onset was nearly always at night, within two or three hours of bedtime. I have never been able to satisfy myself as to an exciting cause. An attack was generally attributed to one of those things that happen so frequently as to make them possibly mere coincidences, *e.g.* worry, excitement, a cold wind, a hot day, fatigue, some food that might not have agreed, being in a crowded room, or going upstairs. Of course, some such event may have been a determining cause, but with one of my patients I was able to eliminate most of these causes. He had a lift to take him upstairs, his diet was most carefully regulated, he never went into a crowded room, but still the attacks recurred. I thought at first that avoiding the stairs had done good ; certainly for six months after the lift was installed he kept well, but eventually the paroxysms returned.

Two of my patients were men, two women. The youngest was fifty-four, the others sixty-eight, seventy-three and seventy at the time of their death. The youngest was a woman who no doubt drank a good deal more than was good for her ; she had a high tension pulse, a trace of albumin in her urine, and I thought suffered from pseudo-angina. I saw her during her first attack, which cleared off quickly. A month later she had another ; and although the lungs cleared up quickly, her general condition was not good. She was weak and her pulse continued too fast. Ten days after her second attack I examined her chest ; the lungs were then quite clear of adventitious sounds, and she seemed to be decidedly better. I had not left the house two minutes when a messenger ran after me. I returned at once, and found her struggling for breath, noisy râles all over the chest, with free expectoration of watery fluid. She became deeply cyanosed, and in two hours died apparently from heart failure.

The other female patient, aged seventy, was the hard-working wife of a labourer. She had had mitral incompetence for some six months, had marked thickening of her arteries, and no albumin in her urine. She had typical attacks at intervals of a few weeks, but after a few months became depressed and drowned herself.

A man of sixty-eight, whose physical signs were an apex beat displaced to the nipple line, thickened arteries, and a pulse tension of 180 had a good deal of dyspnœa and palpitation on exertion, and was a heavy smoker and eater, but temperate in his drink. He had a history of what I thought was an attack of angina. Two paroxysms of œdema occurred at an interval of about a month. I did not see him during the second seizure, as he died before I could get to him, but from the account I received I had no doubt of its nature.

My longest case was a man of seventy-one. He had led a temperate and healthy life, but for two or three years had shown various evidences of arterial degeneration, two slight cerebral hemorrhages and rapidly-failing memory. His pulse tension was only 140, urine normal; he suffered from osteo-arthritis of one hip. For two years his attacks recurred at quite irregular intervals, and he died eventually of heart failure after an attack of shingles. In this case the attacks were followed by dulness over the lower half of the right chest, which persisted for some time, one week to two or three weeks; air appeared to enter less freely over this area, but there were very few râles.

I said that paroxysmal œdema was a better name than acute œdema, as it is necessary to distinguish these conditions from the acute œdema of pneumonia, influenza, advanced mitral disease, dilated heart and Bright's disease, often acute in onset but persisting.

It bears a curious resemblance to cardiac asthma in its mode of onset and in the general appearance of the patient during a paroxysm, but the state of the patient is much more alarming; there is serous effusion into the bronchi, and there is not necessarily other definite evidence of cardiac incompetence.

Various theories have been advanced as to the mechanism of an attack. The one that most appeals to me is that the left ventricle fails to contract properly, so that with every beat it is passing on less blood than the right, the consequence is that pulmonary vessels very rapidly become engorged. Professor Welch produced acute œdema in a rabbit's lungs by squeezing the left ventricle and so paralysing it.

This preliminary failure of the left ventricle becomes evident clinically in the pallor of the patient, the cold skin, the apprehension of death and the small rapid pulse, and the engorgement of the pulmonary vessels by the warning cough and the blood-stained serum that is early expectorated.

This alone does not explain the onset or the cause of an attack. Whether the ventricle fails from some undue strain or from disease of some branch of the coronary artery, or some affection of the auriculo-ventricular bundle, whether the œdema that so rapidly follows the congestion is due to disease of the vessel walls, of the bronchi or alveoli, or to an unhealthy blood state, or to both, there is not as yet enough evidence to prove. It may be that the recurrence of the seizure is due to an accumulating toxin. It may be that rapid breathing, which accompanies the nervous onset at the moment the left ventricle fails, produces a state of deficient carbon dioxide in the blood, a state which would favour the rapid exudation of fluid from the plasma to the tissues or the bronchial tubes. This exuding fluid would prevent the further exhalation of carbon dioxide and soon cause it to be increased in quantity in the blood, and so stimulate the respiratory centre to increased respiration; but now, the lungs being water-logged, this increased respiration is of little avail, the carbon dioxide cannot escape, and the oxygen cannot be absorbed. I only suggest this hypothesis for the consideration of those better able to criticise. My aim is merely to present a clinical record. To complete this I must say a few words about treatment. Nitrites in any form I found useless; and if the theory of failing left ventricle is correct, this is what one would expect, for the nitrite would not alter the ratio between the work of the right and left ventricles. Large hot fomentations no doubt gave some relief; they were soothing, but I do not think that they cut short the attack. I found no evidence of the value of stimulants. Oxygen given freely—it must be freely—gave decided relief, and I think shortened the attack; but nothing to my mind gave so much relief as a hypodermic injection of morphia and atropine. I have only used it in one case, but there it certainly acted like a charm when given soon enough. Since using it for this patient I have regretted that I did not use it before. Whether it acts by quieting the patient's nervous state or by direct action on the heart, I will not venture to say.

